# Investigating Indicators to Assess and Support Alcohol Taxation Policy: Results From the International Alcohol Control (IAC) Study

**DOI:** 10.34172/ijhpm.8551

**Published:** 2025-03-30

**Authors:** Sally Casswell, Karl Parker, Steve Randerson, Taisia Huckle, Lathika Athauda, Aravind Banavaram, Sarah Callinan, Orfhlaith Campbell, Surasak Chaiyasong, Song Dearak, Laura Romero-Garcia, Gopalkrishna Gururaj, Romtawan Kalapat, Khem Karki, Thomas Karlsson, Mom Kong, Shiwei Liu, Norman Maldonado, Juan Felipe González-Mejía, Tim Naimi, Keitseope Nthomang, Opeyemi Oladunni, Kwame Owino, Juan Herrera-Palacio, Phasith Phatchana, Pranil Pradhan, Ingeborg Rossow, Gillian Shorter, Vanlounny Sibounheuang, Mindaugas Štelemėkas, Dao The Son, Kate Vallance, Wim van Dalen, Ashley Wettlaufer, Arianne Zamora

**Affiliations:** ^1^SHORE & Whariki Research Centre, College of Health, Massey University, Auckland, New Zealand.; ^2^Department of Public Health, Faculty of Medicine, University of Kelaniya, Ragama, Sri Lanka.; ^3^3 Department of Epidemiology, National Institute of Mental Health and Neuro Sciences, Bangalore, India.; ^4^Centre for Alcohol Policy Research (CAPR), School of Psychology and Public Health, La Trobe University, Melbourne, VIC, Australia.; ^5^Queen’s University Belfast, Belfast, UK.; ^6^Faculty of Pharmacy, Mahasarakham University (MSU), Kham Riang, Thailand.; ^7^Cambodia Movement for Health, Phnom Penh, Cambodia.; ^8^PROESA, Universidad Icesi, Cali, Colombia.; ^9^National Institute of Mental Health and Neurosciences, Kamataka, India.; ^10^Department of Community Medicine and Public Health, Institute of Medicine, Kathmandu, Nepal.; ^11^Finnish Institute for Health and Welfare, Helsinki, Finland.; ^12^Chinese Center for Disease Control and Prevention, Beijing, China.; ^13^Canadian Institute for Substance Use Research, University of Victoria, Victoria, BC, Canada.; ^14^Department of Social Work, University of Botswana, Gaborone, Botswana.; ^15^Department of Public Health, Adeleke University, Ede, Nigeria.; ^16^Institute of Economic Affairs, Nairobi, Kenya.; ^17^Norwegian Institute of Public Health, Oslo, Norway.; ^18^Faculty of Pharmacy, University of Health Sciences, Vientiane, Lao People’s Democratic Republic.; ^19^Lithuanian University of Health Sciences, Kaunas, Lithuania.; ^20^Thuongmai University, Hanoi, Vietnam.; ^21^Dutch Institute for Alcohol Policy STAP, Utrecht, The Netherlands.; ^22^Centre for Addiction and Mental Health, Toronto, ON, Canada.; ^23^EpiMetrics.Inc, Manila, Philippines.

**Keywords:** Alcohol Pricing Policy, Taxation Methods, Tax/Price Share, Affordability, IAC Study

## Abstract

Alcohol taxation is a key policy to reduce consumption and alcohol harm but evidence on tax design and indicators to assess taxation policy are lacking. Tax design and two indicators: tax as a share of lowest retail price and affordability, were investigated in eight high-income and nine middle-income jurisdictions. Collaborators populated the International Alcohol Control (IAC) study online Alcohol Policy Tool, providing measures of tax design, tax rates; and typical lowest prices available for retail take-away alcohol. These data were used to calculate tax/share of retail price. Affordability of alcohol was assessed against gross national income (GNI) per capita. High-income jurisdictions had higher tax/share and higher affordability on average compared with middle-income jurisdictions. Over the sample as a whole there was no association between these two indicators of tax policy. The tax designs used also varied with high-income jurisdictions more likely to use specific excise tax reflecting potency and middle-income jurisdictions more likely to utilise ad valorem and specific volume based taxes and to use more than one method across a beverage. Increased alcohol taxation to reduce alcohol consumption and harm is established as a high impact policy and is believed to work by affecting affordability. However, less is known about the best taxation methods to reduce affordability or the best measures to monitor and compare alcohol taxation between countries and over time. In this sample of high- and middle-income jurisdictions tax/price share was not found to predict affordability, suggesting the need to further research indicators of alcohol affordability.

## Background

###  Alcohol Taxation

 Taxation of health harming products is an effective policy to reduce harm because higher prices from higher taxes drive down consumption and affect quitting and initiation.^1^ The value of pricing policy to reduce alcohol consumption and harm is well established^2,3^ and taxation is acknowledged as a high impact policy in the Global Alcohol Action plan by the World Health Organization (WHO).^4^ A focus on increasing taxation to increase price is a key element in support provided to countries by WHO, civil society and philanthropy.^5,6^

 In 2018 WHO reported 95% of countries had some form of alcohol taxation in place; however, “many countries have difﬁculty setting an effective tax rate—a tax rate that will lead alcohol consumers to make choices about amounts and forms of alcohol to drink in a way that reduces harm. Decisions on consumption are made against a background of continually varying prices and incomes and other environmental changes that affect the costs of alcohol” (p. 108).^7^ Furthermore, in many jurisdictions, institutional or regulator arrangements that have arisen over time are difficult to disrupt. Existing tax systems reflect a mixture of tradition, pragmatism, industry interests, health policy activism, and protectionism.

 The heterogeneity of products and contexts in which alcoholic drinks are sold and consumed and the possibility of cross substitution make comparisons of taxation policies across countries complex.^2^ Compared with tobacco there is a wider range of products on sale including different beverages and brands. There is also less published research in relation to alcohol tax design and policy development compared with tobacco and this is especially true in low- and middle-income country (LMIC) settings.^8^ Given the value of taxation in reducing use of tobacco^9^ and alcohol^2^ this is an important gap.

 This paper considers three potential indicators of tax and pricing policy strength: alcohol tax as a share of price (tax/price share), affordability and tax design, with data provided by 21 jurisdictions as part of the International Alcohol Control (IAC) study.^10^ Since affordability is established as a driver of consumption, the relationship between tax design and tax as share of price is investigated. As there is evidence the heavier drinkers tend to consume lower priced products,^2^ the typical lowest price is used in this analysis.

 Examining measures used to assess the different elements of tax and price and investigating the relationship between them in a cross-country comparison are intended to inform the development of indicators that will allow monitoring of effective alcohol taxation and pricing policy. Having agreed indicators supports a focus on “what gets measured gets done.”^11^

###  Tax as Share of Price 

 Taxation works as a health policy by affecting the retail price of the targeted products and the measure of tax as a proportion of the retail price has been explored as a public health indicator in relation to alcohol. An alcohol specific excise tax results in a change in relative prices (alcohol vs other goods) and therefore affects consumer behaviour differently than, say, an increase in value added tax (VAT), which affects most or all goods/services.

 Tax is generally passed through to the price,^12^ however analysis of price data in the United Kingdom suggests alcohol retailers may respond to increases in alcohol tax by undershifting their cheaper products (raising prices below the level of the tax increase) and overshifting their more expensive products (raising prices beyond the level of the tax increase). This is likely to impact negatively on tax policy effectiveness because high-risk groups favour cheaper alcohol and thus undershifting is likely to produce smaller consumption reductions.^13^

 Another challenge to the use of tax/price share as an indicator of the strength of alcohol tax policy is that in conditions of increasing income, especially in the context of LMICs with rapidly expanding economies,^14^ and where both excise tax and price remain low, the tax/price share measure is not an adequate measure of the value of excise tax for health.

 In the tobacco field, however, this indicator has long been built into global monitoring^15^ and, as part of WHO recommendations such as MPOWER, is recommended to comprise 75% of total retail price.^9^ The proportion of the retail price of alcohol comprised of excise tax is therefore an indicator worthy of further investigation.

###  Affordability

 A key goal of taxation policy to improve health outcomes is reducing the affordability of alcohol products. Affordability to the consumer is not only affected by taxation and price but also by changes in income and inflation. Alcohol products have become more affordable in most countries. A study of trends in beer prices and affordability (based on per capita gross domestic product) from 1999 to 2016 in up to 92 countries found affordability had increased in most high-income countries (HICs) and LMICs over this time period, and while prices were similar in HIC and LMICs, affordability was higher in HICs.^16^ In the US dramatic increases in affordability occurred particularly in the 1960s and 1970s.^17^ A recent study in Latin American countries found affordability of beer remained unchanged in Argentina, Brazil, Chile, Costa Rica, and Uruguay, dropped in Mexico, and increased only in Colombia and Ecuador.^18^

 Attention has turned to affordability as key in evaluating the outcome of taxation policy and affordability has been linked to changing consumption. A report from the Rand Corporation in 2009^19^ found, despite widespread taxation of alcohol products, alcohol had become much more affordable in all the countries of the European Union (EU) since the mid-1990s and increased consumption and related harm could be attributed to this. Affordability in New Zealand in the years 1985–2011 was more important than real price in determining consumption of alcohol^7^ and affordability has been shown to be associated with decreased alcohol per capita consumption in the Baltic countries and Poland.^20^ However, there is no established criterion for comparing countries in terms of affordability.

###  Tax Design 

 In addition to tax rates different tax designs can affect policy outcomes. A range of alcohol tax designs are seen in different jurisdictions. Excise tax is the most common tax policy to address harm from alcohol (as opposed to import tariffs and sales taxes). However, within excise taxes there are different approaches. Ad valorem taxation is set as a percentage and based on the value of the alcohol product in terms of producer, wholesale or retail price. Specific taxation is based on the ethanol content or the volume of the beverage, whatever its alcohol concentration.^2^

 The use of ad valorem and specific taxation may differ by beverage within country. For example, wine most often has volume based specific taxation applied with the justification of alcohol content being difficult to assess precisely^21,22^ and unitary taxation is mandated for wine in the EU.^23^ After unitary specific taxation wine is frequently taxed by ad valorem.^24^ Wine is also the alcoholic beverage most likely not to be taxed at all, especially in wine producing countries.^18,25^ Spirits are usually taxed at a much higher rate than beer or wine as spirits are cheaper to produce and so higher tax is needed to keep them relatively expensive. There may also be concern that acute harm is more likely from higher potency beverages.^2^

 A tax system based solely on specific tax reflecting the ethanol contained in a beverage has been suggested to be the best option for public health purposes as the amount of alcohol consumed is closely linked to the extent of harm caused. However, specific tax on volume may be easier where administrative resources are less and can affect the price of cheaper beverages.^22^ Ad valorem tax may be subject to manipulation by producers as the tax falls if they reduce the price of the product. However, Thailand, for example, has utilised a mix of specific tax and ad valorem with the objective of protecting abstention. Ad valorem tax increases the price of expensive global brands, which are often the preferred beverage of young drinkers.^2^

 Erosion of the effect of specific excise tax occurs because of inflation and some tax systems recognise this by regular adjustment. However, globally in 2023 less than one in four countries applied an automatically adjusted specific excise tax (p. 16).^22^ Of the 94% of countries in the Americas that apply excise taxes on alcohol products, only a third of these are inflation adjusted.^18^ Inflation adjustment does not always protect against increasing affordability. In New Zealand, for example, despite annual adjustments for inflation, in the context of a low inflation economy but increasing incomes, affordability of alcohol increased,^7^ thus reinforcing the need for governments to pay attention to both inflation and income growth.

###  The International Alcohol Control Study 

 The IAC study has developed the IAC Alcohol Policy Tool to gather data on several of the most effective alcohol policies, and from this calculated an IAC Policy Index reflecting evidence of the strength of the policy domain and its association with alcohol per capita.^26^ The IAC Policy Index has been shown to be associated with abstention,^27^ self-reported drinking patterns,^28^ and to provide a useful framework to monitor alcohol policy change over time within a country.^29^

 The data collected as part of an IAC collaboration in 2022 to 2023 included the tax design, tax rates, and lowest retail prices from off-license (take away) retail outlets.

 The jurisdictions included in the IAC collaboration were a convenience sample: 13 middle-income jurisdictions were included based on funding availability from philanthropic, development agencies and WHO, and reflected likely increasing alcohol consumption and harm; eight high-income jurisdictions were included without specific funding, reflecting established collaborations. These were analysed to provide a descriptive overview and investigate the relationship between three potential indicators of alcohol taxation: alcohol tax as share of price, affordability, and tax design in a range of diverse settings. These data and comparison between jurisdictions provide a framework for localised data collection and interpretation given the importance for policy development.^8^

## Methods

 The design of the study was descriptive, and data were drawn from a convenience sample of 21 countries including both high- and middle-income jurisdictions. Thirteen middle-income countries/jurisdictions from Asia and Africa participated as follows: Thailand, Nepal, Sri Lanka, India (one state Karnataka), Bhutan, Vietnam, Colombia, Cambodia, China, Philippines, Nigeria, Kenya, and Botswana. Eight HICs/jurisdictions participated and these were Canada, Australia, Republic of Ireland, Netherlands, New Zealand, Norway, Finland, and Lithuania. Data were collected from each province and territory of Canada and summarised reflecting population distribution to create a country score.

###  Data Collection

 The online IAC Alcohol Policy Tool was populated with data to assess the stringency of tax design and tax rates and lowest price of beer, wine and spirits available. Data were sourced by collaborators in-country and reflected the prices and tax designs within the period April 2022 to May 2023.

 Tax data (design and tax rates) were collected from government documentation with follow-up interviews with government officials where necessary.

 Most countries sourced lowest alcohol prices from price surveys of a random selection of outlets or outlet observation (where collaborators visited outlets to observe and document prices). In some locations where only a small number of outlets could be directly observed, either online store prices or key informant interviews with relevant officials and community members were used to validate prices. Ireland and Canada sourced prices from alcohol shops and supermarket websites and the websites of state-owned monopolies. In some cases, lowest prices were sourced from national statistics offices (Lithuania), and data from Meituan Instashopping retail software with 678 million users (China).^30^

 For price, countries collected the “typical lowest price in local currency” for beer, wine and spirits in off-premises (for takeaway retail). Prices were compiled in-country from commonly utilised off-premises outlets for beer, wine and spirits, and then averaged across the various establishments. Lowest price was used as it may be easier to assess than typical mid-price, and there is an association between heavier drinking and choice of lower price.^31^

 The research team undertook validation of tax and price data. Verification of tax design and rates involved cross-referencing with official government sources. Price data validation consisted of comparing prices with online listings from alcohol retailers and supermarkets within the countries. In cases where discrepancies arose, we engaged with collaborators to confirm or amend the prices.

###  Measures 

 Tax rates were collected for beer, wine and spirits, which represented the largest proportions of recorded beverages across all countries.^32^ Some LMIC countries have substantial unrecorded alcohol markets, but these beverages are untaxed and therefore excluded. Countries entered tax rates for beer, wine and spirits for ad valorem, tax by volume of beverage or tax by volume of ethanol, and a combination (at least two different tax methods applied at the same time) of these by beverage. Tax design is described as ad valorem, specific excise tax by ethanol and by volume, and a combination of these by beverage.

 Prices in off-premise retail outlets were transformed into purchasing power parity (PPP)^33^ to allow international comparisons^34^ and reported by 15 ml of ethanol.

###  Analysis

####  Purchasing Power Parity 

 Price was calculated for 15 mL of alcohol based on beer containing 5%, wine 12% and spirits 40% (34% in the case of Sri Lanka) abv. A weighted average was used based on the market share of each beverage and then converted to PPP to allow cross-country comparisons.

####  Tax as a Proportion of Price (Tax/Price Share):

 The alcohol tax of each of the three beverages was calculated in the local currency using the supplied tax rates and method, based on the abvs mentioned above, for a standard container size. For ad valorem on retail price, the amount of tax is calculated by multiplying the tax rate by price (excluding VAT/goods and service tax [GST]). When the ad valorem tax base is the wholesale price, margins were assumed to be 20%.^35^ For tax by volume of beverage, the tax per litre was multiplied by the beverage container size. For tax by volume of ethanol, tax amount was derived by multiplying the tax per litre of ethanol by the beverage container size and the abv. We then divided by the lowest price excluding GST/VAT to give the percentage of specific alcohol tax for each beverage. We used a weighted average as above to arrive at a number that represented the average alcohol tax share for the lowest priced beverages within a country.

####  Affordability

 Affordability was calculated based on price (PPP) and gross national income (GNI) per capita^34^ and expressed as the percentage of annual income required to purchase 100 drinks containing 15 ml of ethanol. This was calculated separately for beer, wine and spirits and also weighted by the proportion each beverage contributed to the alcohol market, taken from the Global Information System on Alcohol and Health.^32^

###  Analysis of Associations 

 Pearson correlation coefficients were used to find the strength of the relationships between the variables and were performed in Excel. T-scores were then calculated along with *P* values to determine significance. Plots were created in R (version 4.3.2) using the ggplot2 library.

 The correlations between two sets of variables were calculated: (1) Tax share and Price in PPP and (2) Tax share and affordability (the price in PPP divided by GNI × 100).

## Results

 Price (PPP) of 15 mL of ethanol reflecting the three beverages proportionate to market share varied by jurisdiction. There was more consistency among high-income jurisdictions, but Australia and Netherlands have lower PPP prices. Of the middle-income jurisdictions China’s prices were the lowest, expressed as PPP, and Nepal and Lao PDR the highest.

 Tax is reported as a percentage of typical lowest price (Table 1). Tax/price share was generally higher in high-income jurisdictions (a mean of 47.8%) with the exception of Sri Lanka and Karnataka, which shared the highest tax/price share in the sample with Ireland. Canada had a relatively low tax/price share among the high-income jurisdictions, especially for spirits. Nigeria, and China were relatively low among middle-income jurisdictions (a mean of 36.1%).

**Table 1:** Tax as a Percentage of Lowest Pre-GST/VAT Price From Off-License Retail (Including Supermarkets, Alcohol-Specific, and Convenience Stores)

	**All**^a^	**Beer**	**Wine**	**Spirits**
**High-income**				
Norway	60%	59%	54%	77%
Ireland	63%	45%	79%	81%
Australia	46%	52%	23%	89%
Netherlands	34%	27%	19%	86%
Finland	61%	51%	56%	83%
Canada	32%	27%	18%	49%
New Zealand	34%	31%	21%	64%
Lithuania	53%	32%	45%	75%
Middle-income				
China	16%	3%	10%	24%
Botswana	24%	20%	7%	45%
Thailand	29%	47%	51%	21%
Colombia	43%	38%	34%	57%
Vietnam	36%	36%	26%	36%
Philippines	25%	22%	17%	26%
Sri Lanka	63%	25%	16%	66%
Karnataka	63%	41%	12%	76%
Lao PDR	49%	40%	48%	56%
Kenya	26%	37%	22%	16%
Nigeria	12%	5%	21%	21%
Cambodia	30%	30%	30%	30%
Nepal	55%	39%	35%	81%

Abbreviations: GST, goods and service tax; VAT, value added tax; PDR, People’s Democratic Republic; GNI, gross national income. ^a^ Weighted by market share in each country. Countries and jurisdictions are listed in descending order of GNI.

 Affordability reflects the price (PPP) of alcohol products in relation to GNI per capita and is expressed as the percentage of annual income required to purchase 100 15 mL drinks (Table 2). Affordability was relatively similar among high-income jurisdictions but showed a much bigger range among the middle-income jurisdictions. The average alcohol affordability score for high-income jurisdictions was 0.23%, and for middle-income jurisdictions, 8%.

**Table 2:** Affordability Calculated as Lowest Price of 15 mL Ethanol From Off-License Retail (Including Supermarkets, Alcohol-Specific, and Convenience Stores), Expressed as a Percentage of PPP Divided by GNI Per Capita (Lower Percentage = Higher Affordability)

	**All**^a^	**Beer**	**Wine**	**Spirits**
**High-income**				
Norway	0.18%	0.17%	0.18%	0.22%
Ireland	0.14%	0.14%	0.13%	0.15%
Australia	0.15%	0.20%	0.07%	0.20%
Netherlands	0.12%	0.11%	0.16%	0.08%
Finland	0.28%	0.31%	0.26%	0.25%
Canada	0.21%	0.20%	0.24%	0.20%
New Zealand	0.28%	0.26%	0.33%	0.23%
Lithuania	0.46%	0.41%	0.56%	0.48%
Middle-income				
China	0.57%	0.57%	1.48%	0.50%
Botswana	2.97%	3.02%	3.10%	2.72%
Thailand	2.16%	3.21%	6.68%	1.53%
Colombia	1.92%	1.87%	3.28%	2.00%
Vietnam	3.34%	3.54%	4.15%	0.26%
Philippines	3.37%	7.92%	6.09%	1.34%
Sri Lanka	7.16%	13.92%	21.26%	5.41%
Karnataka	7.70%	11.69%	10.56%	5.43%
Lao PDR	13.69%	10.80%	45.80%	15.41%
Kenya	11.56%	13.44%	16.04%	9.38%
Nigeria	7.32%	7.56%	14.98%	5.10%
Cambodia	13.33%	11.33%	20.31%	32.58%
Nepal	29.03%	38.04%	39.61%	13.68%

Abbreviation: GST, goods and service tax; VAT, value added tax; PDR, People’s Democratic Republic; GNI, gross national income; PPP, purchasing power parity. ^a^ Weighted by market share in each country. Countries and jurisdictions are listed in descending order of GNI.

 Tax/price share was associated with the price of beer in high-income jurisdictions; this was the only significant association found with tax/price share and price of beverage. There was no significant correlation between tax/price share and affordability in either high- or middle-income jurisdictions.

 Unitary taxation (specific on volume of beverage) is more likely to be applied to wine in high-income jurisdictions and to a wider range of beverages in middle-income jurisdictions (Figure). Only one high-income government uses the same tax design approach to all beverages (Norway using specific tax on ethanol). Thailand also taxes all beverages with a specific tax on ethanol but applies ad valorem tax as well (More detail on tax method is given in Supplementary file 1).

**Figure:**
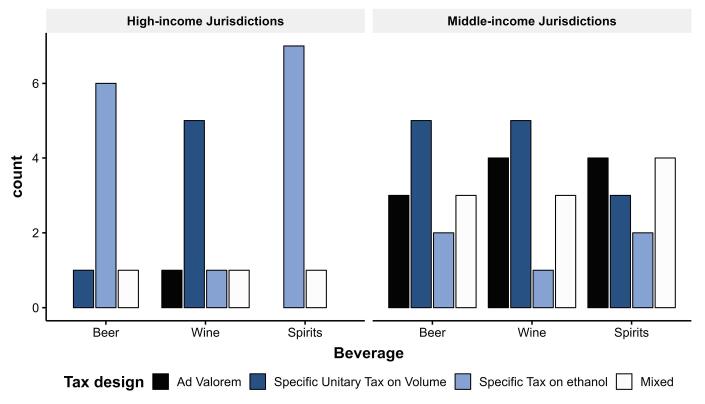
Tax Design by Beverage and Country World Bank Income Classification. Mixed approaches include at least two tax methods from specific tax on volume, specific tax on ethanol, and ad valorem = M.

## Discussion

 Considerable variation was seen across jurisdictions in terms of the tax/price share, as previously reported in an earlier phase of data collection in the IAC collaboration^7^ and as reported in a global review that examined prices of most sold brands.^22^ Tax as a share of retail price as a monitoring and advocacy tool in relation to tobacco control has been recommended at 75% of retail price.^9^ In this sample only three high-income jurisdictions—Norway, Finland, and Ireland, came close to this level, and one middle-income jurisdiction, Sri Lanka. Three of the high-income governments had tax/price shares of about one third in place whereas in five out of 13 of the middle-income jurisdictions, tax/price share was one third or less. Canada’s tax/price share is one of the lower ones at 32%, in line with findings from a previous study undertaken in two large Canadian provinces (in response to claims from vested interests that tax amounted to 50%–80% of price).^36^

 Previous studies estimating alcohol tax/price share have reported shares well below the 75% tobacco aspiration.^9^ Tax on tobacco is more likely to use specific tax whereas there is more use of ad valorem in relation to alcohol. This may reduce the likelihood of reaching a similar tax/price share as tobacco. In the Pan American Health Organization region a regional tax share of 12% across all beverages was reported^24^ and in the European region (EU) alcohol taxes constituted on average only 5·7%, 14·0% and 31·3% of the retail prices of wine, beer, and spirits, respectively. Tax shares were higher in the eastern part of the EU compared to the EU; and some countries in the EU did not have excise taxes on wine.^22,37^

 Tax/price share has previously been reported to be highest for spirits in the Pan American Health Organization region^24^ and this was generally true in the current sample. This is expected as a greater share of tax is required to keep spirits prices in line with other beverages as spirits are cheaper to produce.

 Tax/price share has become a recognised indicator of the policy process (setting tax rates) in tobacco control and it is relatively easy to calculate. However, the main policy outcome goal of taxation is to affect affordability.^2^ In this analysis of lowest alcohol prices available in a diverse range of jurisdictions there was no relationship between affordability, assessed as the amount of income required to purchase alcohol, and the tax/price share. In a similar analysis in Europe the expected relationship was found between affordability and spirits tax/price share, but not for wine and beer.^25^ This highlights the need to develop monitoring indicators beyond tax/price share that take into account affordability.

 The design of the tax system was another dimension measured in this study. The use of ad valorem versus specific tax varied between countries and between beverages. However, although often recommended and thought to be relevant to tax policy outcome,^2^ there appeared to be no clear relationship between tax design and affordability as measured in this analysis.

 Affordability was higher in the HICs and ranged between 0.12% of annual income needed to buy 100 drinks in the Netherlands to 0.28% in Finland and New Zealand. For Lithuania, where an increase in alcohol taxes in 2017 was found to have contributed to a decrease in overall male mortality and inequalities in male mortality,^38^ the percentage of income required was the highest of the high-income jurisdictions at 0.46%. Despite Canada’s low tax/price share, they were also in mid-range for affordability among high-income jurisdictions, at 0.20%.

 The percentage of annual income required to purchase alcohol products was much higher in the middle-income jurisdictions, ranging from a low of 0.57% in China to 29.03% in Nepal. The difference between high- and middle-income jurisdictions may reflect the different income dispersions,^39^ which are not adequately captured in the average income measures used, including in the current study.^16^

 Generally, affordability of spirits was in line with other beverages, but in some Asian (Vietnam, Thailand, Philippines, Karnataka, India, and Nepal) and African (Kenya and Nigeria) countries spirits were more affordable. Where wine was a very small proportion of the alcohol market, it was less affordable.

 The lack of congruence between the measures of tax/price share and affordability speaks to the need for more research to identify the relationships between these measures. A scoping review of the influence of evidence on the uptake of health taxes in LMICs emphasised the value of local and contextualised evidence, but that it is often not available. When available it is used to illustrate the value of taxation in policy agenda settings but is not well used in the technical design or implementation of policy.^8^

###  Limitations

 Use of only off-license price data reflected the greater difficulty of collecting prices from on-premise venues. However, in most jurisdictions sales from off-premises contributed at least 75% to the overall market.

 Most jurisdictions relied on small scale surveys of outlet prices or outlet observation, which may limit generalisability Four jurisdictions used methods other than surveys of outlets to obtain the lowest available price. Two countries (Canada and Ireland) searched online sources from alcohol retailers and supermarkets and two others (China and Lithuania) used already existing sources of data. If this meant that the lowest price available was not obtained, it could have affected results. For three of these four countries this would have the effect of underestimating the percentage of tax/price share. For China, only beer would be underestimated as wine and spirits are taxed ad valorem so the percentage of tax applied is the same no matter the price. The reliance on lowest price is a limitation but this measure was chosen because a typical lowest price may be easier to judge than typical mid price and there is an association between heavier drinking and choice of lower prices. The comparison of countries with very different income distributions may be problematic given no measure of dispersion is included. Given the same price, alcohol is likely to be more affordable in a middle-income country with a relatively equal income distribution than in a country with a similar average level of income but high levels of poverty. Countries may have different distributor margins, which would affect the tax as a percentage of price when ad valorem is based on wholesale price. Abvs for beer and wine may differ from our defaults, although this is unlikely to result in large changes. Spirit prices were requested for 40% abv meaning this issue will not be present with this beverage.

## Conclusion

 Increased alcohol taxation to reduce alcohol consumption and harm is established as a high impact policy. However, less is known about the best taxation methods to reduce affordability, or the best measures to monitor and compare alcohol taxation between countries and over time. In this sample of high- and middle-income countries tax/price share was not found to predict affordability, suggesting the need to further research measures of alcohol affordability.

## Acknowledgements

 We thank Jeff Drope and Chonlathan Visaruthvong for their peer review of the paper.

## Ethical issues

 In the case of the researchers from Massey University, the project has been evaluated by peer review and judged to be low risk. Consequently, it has not been reviewed by one of the University’s Human Ethics Committees. The researcher(s) named in this document are responsible for the ethical conduct of this research.

## Conflicts of interest

 Authors declare that they have no conflicts of interests.

## Supplementary files


Supplementary file 1. Alcohol Excise Taxation Methods by Country and Beverage Type.
