# Supplementary material for: Investigating Indicators to Assess and Support Alcohol Taxation Policy: Results From the International Alcohol Control (IAC) Study
Source: Int J Health Policy Manag. 2025 Mar 30;14:8551. doi: 10.34172/ijhpm.8551 (PMC12257199; doi:10.34172/ijhpm.8551)
Supplement: Supplementary file 1 — Alcohol Excise Taxation Methods by Country and Beverage Type. [file ijhpm-14-8551-s001.pdf]

Article title: Investigating indicators to assess and support alcohol taxation policy: Results from the International Alcohol Control (IAC) study

Journal name: International Journal of Health Policy and Management

Authors' information: Sally Casswell[1]*, Karl Parker[1], Steve Randerson[1], Taisia Huckle[1], Lathika Athauda[2], Aravind Banavaram[3], Sarah Callinan[4], Orfhlaith Campbell[5], Surasak Chaiyasong[6], Song Dearak[7], Laura RomeroGarcia[8], Gopalkrishna Gururaj[9], Romtawan Kalapat[6], Khem Karki[10], Thomas Karlsson[11], Mom Kong[7], Shiwei Liu[12], Norman Maldonado[8], Juan Felipe González-Mejía[7], Tim Naimi[13], Keitseope Nthomang[14], Opeyemi Oladunni[15], Kwame Owino[16], Juan Herrera-Palacio[8], Phasith Phatchana[6], Pranil Pradhan[10], Ingeborg Rossow[17], Gillian Shorter[5], Vanlounny Sibounheuang[18], Mindaugas Štelemėkas[19], Dao The Son[20], Kate Vallance[13], Wim van Dalen[21], Ashley Wettlaufer[22], Arianne Zamora[23]

[1]SHORE & Whariki Research Centre, College of Health, Massey University, Auckland, New Zealand.

[2]Department of Public Health, Faculty of Medicine, University of Kelaniya, Ragama, Sri Lanka.

[3]Department of Epidemiology, National Institute of Mental Health and Neuro Sciences, Bangalore, India.

[4]Centre for Alcohol Policy Research (CAPR), School of Psychology and Public Health, La Trobe University, Melbourne, VIC, Australia.

[5]Queen's University Belfast, Belfast, UK.

[6]Faculty of Pharmacy, Mahasarakham University (MSU), Kham Riang, Thailand.

[7]Cambodia Movement for Health, Phnom Penh, Cambodia.

[8]PROESA, Universidad Icesi, Cali, Colombia.

[9]National Institute of Mental Health and Neurosciences, Kamataka, India.

[10]Department of Community Medicine and Public Health, Institute of Medicine, Kathmandu, Nepal.

[11]Finnish Institute for Health and Welfare, Helsinki, Finland.

[12]Chinese Center for Disease Control and Prevention, Beijing, China.

[13]Canadian Institute for Substance Use Research, University of Victoria, Victoria, BC, Canada.

[14]Department of Social Work, University of Botswana, Gaborone, Botswana.

[15]Department of Public Health, Adeleke University, Ede, Nigeria.

[16]Institute of Economic Affairs, Nairobi, Kenya.

[17]Norwegian Institute of Public Health, Oslo, Norway.

[18]Faculty of Pharmacy, University of Health Sciences, Vientiane, Lao People's Democratic Republic.

[19]Lithuanian University of Health Sciences, Kaunas, Lithuania.

[20]Thuongmai University, Hanoi, Vietnam.

[21]Dutch Institute for Alcohol Policy STAP, Utrecht, The Netherlands.

[22]Centre for Addiction and Mental Health, Toronto, ON, Canada.

[23]EpiMetrics.Inc, Manila, Philippines.

***Correspondence to:** Sally Casswell, s.casswell@massey.ac.nz

Citation: Casswell S, Parker K, Randerson S, et al. Investigating indicators to assess and support alcohol taxation policy: Results from the International Alcohol Control (IAC) study. Int J Health Policy Manag. 2025;13:8551. doi:10.34172/ijhpm.8551

Supplementary file 1. Alcohol Excise Taxation Methods by Country and Beverage Type

Supplementary Table 1: Alcohol excise taxation methods by country and beverage type.

	Beer	Wine	Spirits
**Norway**	Specific Tax on Ethanol	Specific Tax on Ethanol	Specific Tax on Ethanol
**Ireland**	Specific Tax on Ethanol	Specific Unitary Tax on Volume	Specific Tax on Ethanol
**Australia**	Specific Tax on Ethanol	Ad Valorem	Specific Tax on Ethanol
**Netherlands**	Specific Unitary Tax on Volume	Specific Unitary Tax on Volume	Specific Tax on Ethanol
**Finland**	Specific Tax on Ethanol	Specific Unitary Tax on Volume	Specific Tax on Ethanol
**Canada***			
**Ontario**	Specific Unitary Tax on Volume	Ad Valorem<br>Specific Unitary Tax on Volume	Ad Valorem<br>Specific Unitary Tax on Volume<br>Specific Tax on Ethanol
**Quebec**	Specific Unitary Tax on Volume	Specific Unitary Tax on Volume	Specific Unitary Tax on Volume<br>Specific Tax on Ethanol
**Alberta**	Specific Unitary Tax on Volume	Specific Unitary Tax on Volume	Specific Unitary Tax on Volume<br>Specific Tax on Ethanol
**British Columbia**	Ad Valorem<br>Specific Unitary Tax on Volume	Ad Valorem<br>Specific Unitary Tax on Volume	Ad Valorem<br>Specific Tax on Ethanol
**Saskatchewan**	Ad Valorem<br>Specific Unitary Tax on Volume	Ad Valorem<br>Specific Unitary Tax on Volume	Ad Valorem<br>Specific Tax on Ethanol
**Manitoba**	Specific Unitary Tax on Volume	Specific Unitary Tax on Volume	Specific Tax on Ethanol
**Nova Scotia**	Specific Unitary Tax on Volume	Specific Unitary Tax on Volume	Specific Tax on Ethanol
**New Brunswick**	Specific Unitary Tax on Volume	Specific Unitary Tax on Volume	Specific Tax on Ethanol
**Newfoundland and Labrador**	Specific Unitary Tax on Volume	Specific Unitary Tax on Volume	Specific Tax on Ethanol
**Prince Edward Island**	Ad Valorem<br>Specific Unitary Tax on Volume	Ad Valorem<br>Specific Unitary Tax on Volume	Ad Valorem<br>Specific Tax on Ethanol
**Northwest Territories**	Specific Unitary Tax on Volume	Specific Unitary Tax on Volume	Specific Tax on Ethanol
**Nunavut**	Specific Unitary Tax on Volume	Specific Unitary Tax on Volume	Specific Tax on Ethanol
**Yukon**	Ad Valorem<br>Specific Unitary Tax on Volume	Ad Valorem<br>Specific Unitary Tax on Volume	Ad Valorem<br>Specific Tax on Ethanol
**New Zealand**	Specific Tax on Ethanol	Specific Unitary Tax on Volume	Specific Tax on Ethanol
**Lithuania**	Specific Tax on Ethanol	Specific Unitary Tax on Volume	Specific Tax on Ethanol
**China**	Specific Unitary Tax on Volume	Ad Valorem	Ad Valorem
**Botswana**	Specific Tax on Ethanol	Specific Unitary Tax on Volume	Specific Tax on Ethanol
**Thailand**	Ad Valorem<br>Specific Tax on Ethanol	Ad Valorem<br>Specific Tax on Ethanol	Ad Valorem<br>Specific Tax on Ethanol

**Colombia**	Ad Valorem	Ad Valorem<br>Specific Tax on Ethanol	Ad Valorem<br>Specific Tax on Ethanol
**Vietnam**	Ad Valorem	Ad Valorem	Ad Valorem
**Philippines**	Specific Unitary Tax on Volume	Specific Unitary Tax on Volume	Ad Valorem<br>Specific Tax on Ethanol
**Sri Lanka**	Specific Tax on Ethanol	Specific Tax on Ethanol	Specific Tax on Ethanol
**Karnataka**	Ad Valorem<br>Specific Unitary Tax on Volume	Specific Unitary Tax on Volume	Specific Unitary Tax on Volume
**Lao PDR**	Ad Valorem	Ad Valorem	Ad Valorem
**Kenya**	Specific Unitary Tax on Volume	Specific Unitary Tax on Volume	Specific Unitary Tax on Volume
**Nigeria**	Ad Valorem<br>Specific Unitary Tax on Volume	Ad Valorem<br>Specific Unitary Tax on Volume	Ad Valorem<br>Specific Unitary Tax on Volume
**Cambodia**	Ad Valorem	Ad Valorem	Ad Valorem
**Nepal**	Specific Unitary Tax on Volume	Specific Unitary Tax on Volume	Specific Unitary Tax on Volume

*Scores for Canada were combined using a weighted average based on the population of each province/territory.
